# Alliance: a common factor of psychotherapy modeled by structural theory

**DOI:** 10.3389/fpsyg.2015.00421

**Published:** 2015-04-21

**Authors:** Wolfgang Tschacher, Hermann Haken, Miriam Kyselo

**Affiliations:** ^1^Universitätsklinik für Psychiatrie und Psychotherapie, Universität BernBern, Switzerland; ^2^Center of Synergetics, Institut für Theoretische Physik, Universität StuttgartStuttgart, Germany; ^3^Department of Theoretical Philosophy, Vrije Universiteit AmsterdamAmsterdam, Netherlands

**Keywords:** psychotherapy, common factors, synergetics, attractor dynamics, enactive cognitive science, mathematical psychology, relaxation times, Fokker-Planck equation

## Abstract

There is broad consensus that the therapeutic alliance constitutes a core common factor for all modalities of psychotherapy. Meta-analyses corroborated that alliance, as it emerges from therapeutic process, is a significant predictor of therapy outcome. Psychotherapy process is traditionally described and explored using two categorically different approaches, the experiential (first-person) perspective and the behavioral (third-person) perspective. We propose to add to this duality a third, structural approach. Dynamical systems theory and synergetics on the one hand and enactivist theory on the other together can provide this structural approach, which contributes in specific ways to a clarification of the alliance factor. Systems theory offers concepts and tools for the modeling of the individual self and, building on this, of alliance processes. In the enactive perspective, the self is conceived as a socially enacted autonomous system that strives to maintain identity by observing a two-fold goal: to exist as an individual self in its own right (distinction) while also being open to others (participation). Using this conceptualization, we formalized the therapeutic alliance as a phase space whose potential minima (attractors) can be shifted by the therapist to approximate therapy goals. This mathematical formalization is derived from probability theory and synergetics. We draw the conclusion that structural theory provides powerful tools for the modeling of how therapeutic change is staged by the formation, utilization, and dissolution of the therapeutic alliance. In addition, we point out novel testable hypotheses and future applications.

## Introduction

### Common factors in psychotherapy

In this theoretical and methodological paper, we wish to address the therapeutic alliance, an essential constituent of psychotherapy, in a novel way. We will start from the common factors debate in psychotherapy research, which has focused on alliance as a prominent common factor of psychotherapy process. An important, albeit often tacit, issue in psychotherapy and in cognitive science is the persisting duality of experiential (first-person) and behavioral (third-person) approaches in investigations of psychological entities such as the therapeutic alliance. Each common factor, each active ingredient of psychotherapy, can be defined and monitored either at the experiential or at the behavioral level.

A basic definition of psychotherapy may be this: Psychotherapy is a learning process, which relies on general mechanisms of action that are implemented using psychological techniques and interventions. Techniques are provided during a scheduled social interaction between therapist and patient with the goal of alleviating the patient's disorders and improving health and well-being.

Psychotherapy research has shown a sequence of developmental stages since its beginnings in the early 19th century (Freud, 1900/1989). A large number of modalities of psychotherapy have evolved since Freud's psychoanalysis: humanistic, (cognitive-) behavioral, and systemic schools. In the 1950s, the field entered its “legitimation phase” because general concerns had been prominently voiced that psychotherapy effectiveness would not exceed spontaneous remission rates. In response to this criticism, a vast number of efficacy studies, reviews, and meta-analyses were conducted; they demonstrated that psychotherapies of many different approaches are at least moderately, often highly effective for the treatment of many different kinds of psychopathology. The field thus arrived at a firm evidence-based consensus that psychotherapy is considerably more effective than no treatment or placebo treatment. One result of the legitimation phase was however unexpected: all meta-analyses consistently found only few signs of a superiority of any particular one of the many psychotherapy modalities (Wampold, 2001). As a consequence, a novel research priority developed, aimed at identifying the *common factors* that apparently underlie all psychotherapies. The field entered a new stage with a focus on the psychotherapy process in general: What is it that makes therapy effective?

The current discussion in psychotherapy research strongly emphasizes these common factors. Such factors are called “common” for two reasons: First, they are present in quite different and even opposing therapy modalities. A second meaning of “common” is that these factors are effective in the treatment of quite different disorders and problems. In the discussion on the general mechanisms of how psychotherapy works, common (i.e., unspecific) factors are usually contrasted with specific factors that are grounded in the therapist's specific techniques and interventions. Proponents of the common factors view would assume that the common factors, not the specific techniques, are crucially or even exclusively involved in bringing about psychotherapy effects. Examples of common factors that are shared by all psychotherapy modalities are “hope instilled in the patient,” the “cognitive restructuring” of the patient's belief system, the patient's “corrective emotional experience,” and patient and therapist establishing a trusting, cooperative relationship, namely a “therapeutic alliance.” A survey-based description of those 22 common factors that have been discussed most frequently was provided by Tschacher et al. (2014b).

Many of the common factors put forward by psychotherapy researchers have overlapping content. Therefore, several authors have tried to systematize the discussion by grouping the common factors. One may distinguish (Cornsweet, 1983) aspects of the therapeutic relationship, cognitive factors and therapist factors. Omer and London (1989) clustered common factors in four higher-level categories: Relationship factors, expectancy effects, reorganizing factors, and therapeutic impact. Grencavage and Norcross (1990) found in their review of the literature five groups of common factors: Client characteristics, therapist qualities, change processes, treatment structure, and therapeutic relationship. Grawe (2004) reviewed the empirical evidence of psychotherapy research accumulated toward the end of the 20th century, and proposed four essential common factors: Activation of the problem in the therapeutic setting; activation of a patient's resources; motivational clarification; coping with the problem. Finally, factor analysis of the mentioned list of 22 common factors (Tschacher et al., 2014b) resulted in four dimensions: Patient's cognitive processing, problem solving, emotional processing, and the building up of a therapeutic alliance.

The common factor that is, sometimes implicitly, present in all of these groupings, and that was investigated in most detail is clearly the therapeutic relationship or *alliance*; the general importance of alliance as a change factor is recognized by almost all researchers of psychotherapy process. By the mid-1990s, a majority of empirical studies had supported a positive effect of alliance on outcome, compared to some studies finding no association and a single study finding a negative association. This clear evidence in favor of the contribution of alliance was strengthened by additional empirical studies one decade later (Orlinsky et al., 2004).

Alliance, as it is discussed in psychotherapy research, is itself composed of a number of variables. An encompassing meta-analytic review of the effectiveness of therapeutic relationships was conducted in a task force project of the American Psychological Association to establish an evidence base for therapy relationships (Norcross, 2011). The following aspects of therapeutic relationships were distinguished: Bond, empathy, goal consensus, positive regard, congruence, collecting feedback from the patient, repairing relationship ruptures, avoiding countertransference, and matching the individual patient. In sum, each of these aspects of a multi-faceted alliance construct positively and significantly predicts therapy outcome. The respective meta-analyses yielded effect sizes around 0.3, i.e., a weak to moderate effect of alliance with respect to therapy success.

### The duality of perspectives on alliance

**Figure 1:**
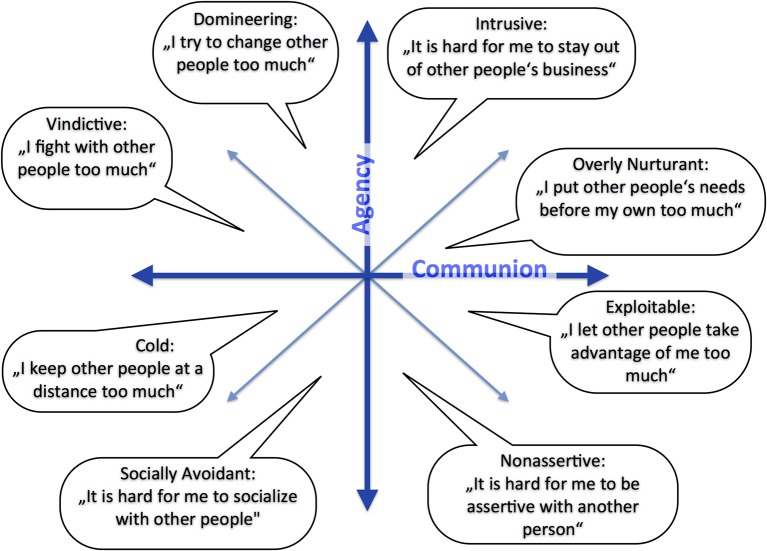
**Circumplex model of interpersonal experiences and problems**. The description of a problem is given for each octant of the circumplex (after Horowitz, 2004).

In psychotherapy research, alliance is addressed at two categorically different levels. From a *first-person perspective*, alliance is something that is *experienced* by the members of the relationship. A common representation of interpersonal experiences in psychology is the interpersonal circumplex model (Leary, 1957). Interpersonal space is assumed to have two dimensions, communion (love) and agency (power). Interactional experiences in a social relationship such as the therapeutic alliance can be described as a blend of experiences of communion and agency, thus as a region in a two-dimensional circumplex (Figure 1). A characterizing property of experiential states is that they have “intentionality” (Brentano, 1874): Experiences are “about something” in the sense that an experience almost always contains a reference to something.

A *third-person perspective* of alliance is accessible by the objective observation of relationship behavior. Behavioral measures are overt body movement, the pressing of buttons in a psychological experiment, physiological responses etc. In addition, there is a standard short-cut in psychology by which even experiential data (i.e., subjective judgments) can be transformed into an objective, third-person form. To do this, experiential judgments are operationalized by intersubjectively validated measures such as questionnaires. This has been done extensively in the context of (experiential) circumplex theory, e.g., by the Inventory of Interpersonal Problems (IIP, Horowitz et al., 1988), a measure of current difficulties in interpersonal functioning. The eight subscales of the IIP pertain to eight octants of the circumplex model of interpersonal behavior (Figure 1), and the respective blend of agency and communion is assessed using questionnaire items, which quantify a person's judgments.

**Figure 2:**
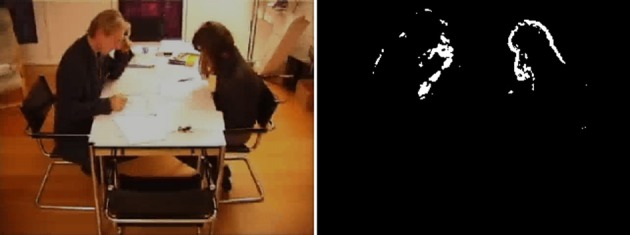
**Movement of interactants in a video (left) can be visualized by highlighting of pixel changes between consecutive frames (right)**. The correlation of both persons' time series of pixel changes yields a measure of non-verbal synchrony.

The physical level of alliance also yields a third-person description; it can be directly addressed through observable motor behavior and may be defined in terms of the degree of behavioral coordination and cooperation of persons. There are a number of implementations of this general idea. A method that assesses such coordination by the quantification of synchronized motor behavior of a patient-therapist dyad is Motion energy analysis (MEA, Ramseyer and Tschacher, 2014; Tschacher et al., 2014a). MEA measures the correlation of body movement of interactants, where movement may be estimated by the amount of pixel changes in a digital video of the respective interaction (Figure 2). The resulting correlational measure yields the degree of non-verbal synchrony, which has been repeatedly shown to be a significant marker of alliance quality.

The idea of objectifying social alliance by a synchrony measure can be generalized to several other fields of social interaction. Synchrony may come to the fore also in the shape of verbal synchrony, such as the interactants' shared use of words, symbols, idioms, or narratives—the higher the overlap in their vocabularies, the closer the alliance. People interacting closely also tend to show similar patterns of facial expressions (such as smiling or frowning), features of the voice (e.g., alignment of prosody), and body postures (e.g., both interactants crossing arms in front of the chest). In social psychology, such phenomena of bodily synchronizations are summarized by the concepts of contagion (Hatfield et al., 1994) or mimicry (Chartrand and Lakin, 2013). At an abstract level, the various synchrony findings indicate that social relationships are not merely experienced but definitely also embodied.

Our goal in this article is to arrive at a more encompassing understanding of the common factor “therapeutic alliance.” According to our hypothesis it is thereby necessary, and viable, to overcome the conventional separation between the subjective-experiential and the objective-behavioral descriptions of relationship. The methodology of operationalization in psychology likewise attempts to overcome this separation by translating experiences into behavioral data. We wish to proceed a step further still, and explore a level that is neither experiential nor behavioral. We demonstrate that there are methodologies by which this may be accomplished: the structural science of systems theory and the enactive approach in embodied cognitive science. Finally, both methodologies will be implemented to elucidate the concept of therapeutic alliance.

## Methodology: beyond duality

### Enactivism

In a previous article, Kyselo (2014) conceptualized the psychological *self* as an autonomous system (Maturana and Varela, 1980) that is socially “co-enacted” and constituted by two poles, distinction and participation. The psychological self, within mental architecture seemingly the core of individuality, is therefore at the same time a social structure. This means that the human self is brought forth through interactions and relations with others and is characterized by two dialectical strivings, on the one hand toward a sense of self as being distinct from others, and on the other hand toward a sense of self as being open to and affected by others. The resulting directions or poles, distinction and participation, shape the individual's relations and interactions. They may be viewed as a fundamental dimension of the self, and at the same time, the building block of sociality and thus, in a next step, social alliance. Maintenance of the self is effected by the intersubjective co-negotiation between distinction and participation.

The enactive approach aims at bridging the gap between the first-person and third-person perspectives described in the previous section. It holds that subjectivity and third-person organizational description are complementary: With the generation of an autonomous identity a system is considered to acquire its very own perspective on the world, based of which it evaluates interactions and constructs meanings (Varela, 1995; Weber and Varela, 2002; Thompson, 2007). Therefore, enactivism transcends the duality of subjective and objective knowledge in the fundamental psychological construct of the self: Objectively speaking, the self is a self-other enacted network of social relations, subjectively speaking this network brings about a sense of self which grounds a basic perspective on the world. Subjective and objective perspectives are thus joined in the lived social existence of the self.

### Dynamical systems theory and synergetics

A second method to mend the duality of perspectives may be seen on the background of the theory of science (Brunner et al., 2011), which differentiates not only the natural sciences (e.g., physics, biology, geology) from the humanities and arts (*Geisteswissenschaften* such as history, science of art, philology), but also recognizes a further group of sciences, the structural sciences (e.g., mathematics, cybernetics, systems theory). Structural sciences reside at a level where only the relations between formal objects are addressed, irrespective of any content or ontology. Correspondingly, research subjects in psychology, including the self and the therapeutic alliance, may be analyzed using a threefold physical-mental-structural view instead of the dualist body-mind dichotomy. This grouping corresponds to Popper's three worlds theory, which distinguishes the physical world, the mental world, and the world of thought products and art works (Popper, 1972).

The general idea is that the structural perspective is neither a first- nor third-person perspective. Nevertheless, experiential or objective data may serve as the components of structural models. In other words, structural models suspend the categorical difference of first- and third-person descriptions. An encompassing structural framework is for instance offered by dynamical systems theory (DST, e.g., Guckenheimer and Holmes, 2002) and self-organization theory (synergetics: Haken, 2000). DST is aimed at the deterministic modeling of change and stability of a system, whereas synergetics specifically models pattern formation and phase transitions, in which deterministic and stochastic processes are combined. Both structural disciplines are based on a formal mathematical terminology.

Proponents of self-organization theory have presented various approaches to model the emergence of novel properties arising from lower levels of a hierarchy. Such “contextual emergence” (Atmanspacher and beim Graben, 2009) assumes a structural relation between different levels of description. For example, the mind may be viewed in the context of neuronal dynamics (Haken and Tschacher, 2010). A recent reconstruction of intentionality, the defining property of first-person experience, in terms of synergetics and contextual emergence was presented by beim Graben (2014). He used a dissipative non-equilibrium system (so-called magnetic surface swimmers) to show that self-assembling patterns with intentional behavior can be based on, but not reduced to, simple physical and electromagnetic laws.

In this vein, Kyselo and Tschacher (2014) have previously elaborated the idea that the psychological self—and social relationships between selves—may be analyzed using structural concepts joining enactive embodied theorizing with DST. As mentioned, the individual self is hypothesized, in enactivist terms, to exhibit two independent strivings, distinction and participation. Kyselo and Tschacher (2014) proposed a model using a geometrical phase space that is spanned by distinction and participation as two different dimensions; they are seen as the coordinate axes of phase space. The stability of all distinction/participation states were symbolized by basins and hills in the “landscape” of such a phase space. The elevation of a state in phase space, its third dimension, reflects an “energetic” value (the potential *V*) of that state. As system states *x* tend to relax toward states of lower energy, the momentary state of a system follows trajectories (like a ball that would roll downhill). In DST, a potential minimum (basin) is an *attractor* of the phase space of a system, denoting states with lower potential and thus higher probability; analogously, a hill denotes a repellor. One may say that the behavior in *x* follows a probability distribution *p*, where *p*(*x*) is negatively related to *V*(*x*).

**Figure 3:**
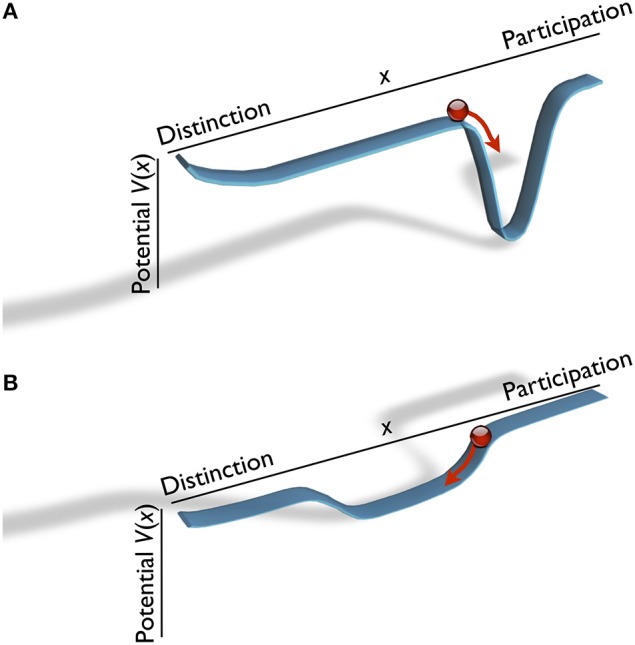
**Two examples of structural models of the phase space of the self of an individual**. The potential function represents repelling and attracting regions of the distinction / participation axis *x*. The current state of the individual is illustrated by the position of the “ball” in the landscape. With time the ball tends to roll “downhill,” into an attractor with low potential values (trajectory: red arrow). Note that when started from identical initial conditions, the self-state changes in opposite directions in **(A,B)**.

Here, we continue the project of describing interaction dynamics in structural terms. We assume that distinction and participation are two poles of a single dimension *x*, akin to Horowitz' communion axis of the circumplex (cf. Figure 1). Compared to Kyselo and Tschacher (2014), who allowed orthogonal (uncorrelated) distinction and participation dimensions, this means a simplified initial step of modeling. Thus we premise here that distinction and participation are mutually exclusive states of *x*. Two examples of the ensuing one-dimensional phase space are depicted in Figure 3. Let us illustrate this for the case of an individual who commonly interacts (behaviorally) “*in sync*” with other individuals, which may (experientially) be related to wishes of signaling social connectedness. In the structural model, this translates to a state of *x* more toward participation. Consequently, the phase space of this individual may have an attractor in a region with rather high participation (Figure 3A). Qualitatively different behavior is represented by the attractor in Figure 3B, which is flatter and broader.

In what follows we will apply this methodological framework—enactive terms integrated in a DST approach—in order to address our main question: How can we use these tools to model and understand the therapeutic alliance?

## Toward a minimal model of therapeutic alliance

We have argued that the individual core of mental architecture, the self, can at the same time be seen as a social structure. In the following, we will rely on the assumption that each individual can be described in terms of the socially enacted self and as obeying a two-fold norm of (social) distinction and (social) participation. From this perspective it is therefore less than surprising that psychotherapeutic intervention, as a learning process acting on the patient's self, is a social project at all levels—the “commonest” of all common factors of psychotherapy is the social alliance between therapist and patient. In Kyselo and Tschacher (2014) we have described a conceptualization of dyadic relationships. Our goal in the present article is to elucidate a particular relationship, namely that between therapist and patient. We thus aim at a minimal model of (dyadic) therapeutic relationships.

Alliance is a system that evolves through the coupling of the individual self-systems of the interactants; such self-systems were depicted in Figure 3. In terms of DST, the therapeutic alliance can be described as a new, joint phase space on the basis of the selves of therapist and patient. Changes in the interaction dynamics of the alliance can be modeled as trajectories through this phase space, and recurring or stable interaction patterns are represented by attractors in it. The individual self-models of Figure 3 consisted of the distinction-participation dimension *x*, and a further dimension, the potential *V*, that gives a value to each *x*. Accordingly, the alliance phase space is constructed by merging two individual phase spaces (the therapist's and the patient's, *x*_1_ and *x*_2_) and a potential value. This three-dimensional structure represents the joint complexity of the alliance in terms of autonomy of two individuals who negotiate their respective identities. The alliance phase space is spanned by *x*_1_, *x*_2_, and the attraction/repulsion of each point in the plane, *V*(*x*_1_, *x*_2_). *V*(*x*_1_, *x*_2_) represents the result of therapist's and patient's negotiations of the alliance between them.

Even if we know the individual phase spaces, they will not suffice to fully specify what the “landscape” of alliance phase space looks like. We cannot know the negotiation results. The structure of the landscape depends on the contributions or behavior of the specific individuals, but their resultant particular interaction dynamics over time is not completely determined by them, instead it emerges from their interaction. This “negotiation” may appear reminiscent of game theory, a mathematical tool used by decision-making theory in economics, biology and linguistics (Benz et al., 2006). Game theory would conceive of alliance formation as the result of two rational players who use linguistic and other strategies to maximize their respective utilities and pay-offs. Some game-theoretical scenarios lead to a Nash equilibrium, which might be viewed as representing the alliance that is formed by the cooperative, competitive, or mixed strategies of the players, therapist and patient. Game theory, however, is fraught with a priori assumptions such as players' rationality and the presence of a defined utility matrix, which appears rather far-fetched in the psychotherapy context. In therapy, one “player,” the therapist, will not pursue his own benefit but the patient's best interests. Furthermore, psychotherapy concerns the clarification of irrational experiences and behaviors.

**Figure 4:**
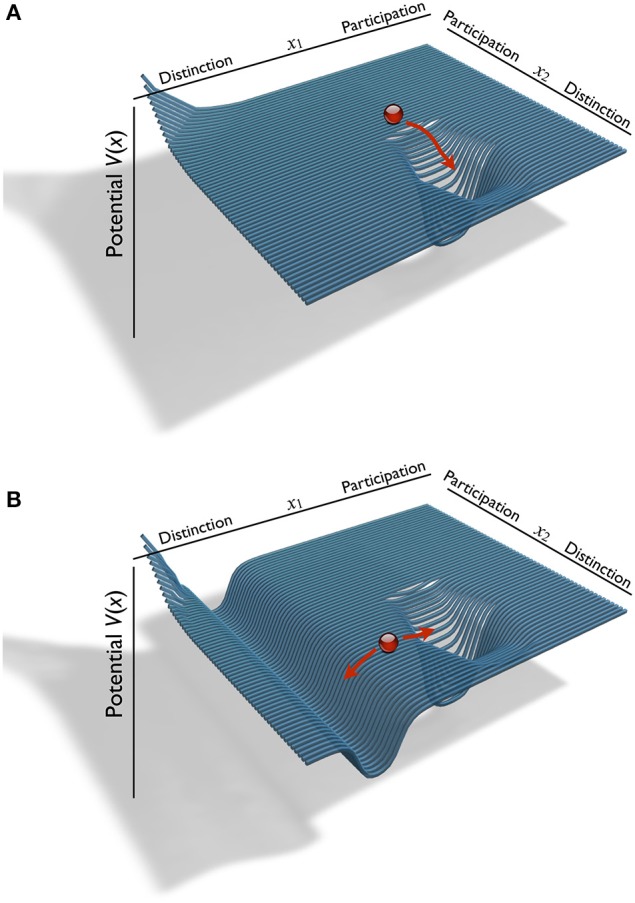
**Two exemplary structural models of the phase space of an alliance**. The potential function *V*(*x*) represents repelling and attracting regions of the distinction/participation plane of therapist and patient, *x*_1_ and *x*_2_ (red arrows: trajectories). **(A)** Phase space with one attractor. **(B)** Two attractors.

Let us continue with our description of alliance phase space. Two possible examples for the formation of an alliance phase space are illustrated in Figure 4A. It shows an alliance with a single attractor, which is located in a region with moderately participatory behavior of person 1 (*x*_1_) and distinctive behavior of person 2 (*x*_2_). A quite different alliance pattern is given by Figure 4B: in addition to the attractor of the other example, person 1's preference for highly distinctive behavior prevails for all values *x*_2_ of the other person. The two participants of this dyad have constructed a one-sided, but quite coherent social system. In the extreme case of dyadic interaction completely lacking, the alliance phase space would be flat, unless where both persons incidentally have overlapping potential basins or hills.

In other words, the formation of an alliance is not merely determined by the phase spaces of the individual persons alone, but critically depends on the kind of coupling between the persons when the alliance is formed. Alliance formation will in turn also influence individual self-maintenance. We may know the shapes of the individual phase spaces and thus their probability distributions, but the joint probability *p*(*x*_1_, *x*_2_) is unknown. In order to shed more light on this process of alliance formation and the emerging joint probability distribution, we will now model the alliance in formal terms.

First we consider the individual person. We assume that his/her self can be described by a potential *V* that depends on a single variable *x*, i.e., *V* = *V*(*x*), which has a single minimum (see the examples in Figure 3). According to our background assumptions, *x* belongs to the interval *x* ⊂ (*x*_distinction_, *x*_participation_). We attach to *V*(*x*) a probability distribution

(1) p(x)=N exp(−V(x)/Q).

*V*(*x*)/Q is an analog to the thermodynamical concept of free energy (Friston, 2011). In Equation (1) the normalization constant N is chosen such that all probabilities add up to 1:

(2) ∫p(x)dx=1,

i.e.,

(3) $$\text{N}={\left(\int \exp\left(-V(x)/\text{Q}\right)dx\right)}^{-1}.$$

Q is a constant parameter (see also below). A simple though plausible model for Equation (1) is a Gaussian distribution,

(4) $$p(x)=\sqrt{\frac{\alpha }{\pi }}\exp\left(-\alpha {\left(x-\beta \right)}^{2}\right)$$

(5) which is centered around x=β

The Gaussian Equation (4) becomes flat for α small (cf. Figure 3B), and strongly peaked for α large (cf. Figure 3A). This invites a psychological interpretation: α large models a “strong” or rigid personality *x*, which is stable against external perturbations. Small α represents an “unstable self” that is easily influenced by outside forces (in terms of social science statistics, α is the inverse of variance, β is the mean of the distribution).

Second, let us turn to an alliance of two individuals 1 and 2, who have distinction-participation variables *x*_1_ and *x*_2_, respectively. In this case it is more convenient to start with the joint probability *p*(*x*_1_, *x*_2_), which we write in analogy to Equation (1) as

(6) $$p(x_{1},x_{2})=N_{1,2}\exp(-V(x_{1},x_{2})/\text{Q})$$

We first consider two limiting cases. The first case is: there is no alliance formation, thus the two selves are independent of each other. According to probability theory, Equation (6) then factorizes, i.e., the joint probability is simply the product of the individual probabilities

(7) $$p(x_{1},x_{2})=p(x_{1})p(x_{2}).$$

Independence in alliance formation may lead to a phase space as in Figure 4A: The attractor is in a region where both persons have *p*(*x*) > 0.

A very strong mutual (symmetric) alliance, on the other hand, implies a strong coupling between *x*_1_ and *x*_2_, which can be modeled by Dirac's (infinitely) peaked δ-function

(8) $$p(x_{1},x_{2})=\delta (x_{1}-x_{2})$$

Our concern in psychotherapy is a moderately to strongly unidirectional alliance in the sense of a bond of the patient with the therapist: the patient's self is “bound” to the therapist. An alliance phase space of this kind may look like in Figure 4B.

To capitalize on relevant results of synergetics, we characterize the behavioral patterns of persons 1 and 2 as follows: Person 1 (the therapist) changes his/her behavior slowly over time (due to the therapist's role behavior, which is rooted in work experience and training) with the limiting case of a therapist who is not subject to any external influences at all:

(9) $$p(x_{1})\approx \delta \left(x_{1}-x_{\text{fixed}}\right)$$

Person 2, the patient, is willing (and able) to change behavior within a certain time. In mathematical terms, these “relaxation times” of behavior change (“relaxation” is unrelated to the psychological meaning of this word—in physical sciences, relaxation time is defined as the time needed by a system to return to equilibrium after a perturbation) are *t*_1_ ≫ *t*_2_. Correspondingly, the relaxation constants γ = 1/*t* obey

(10) $$\gamma_{1}\ll \gamma_{2}.$$

Under these conditions, the slaving principle of synergetics holds (Haken, 1977). The slaving principle states that slowly varying systems achieve control over systems varying more quickly (it should be noted that slaving is a purely technical term not to be confused with political connotations such as “enslavement”). According to this principle, the joint probability *p*(*x*_1_, *x*_2_) can be written, other than in Equation (7), as

(11) $$p(x_{1},x_{2})=p\left(x_{1}|x_{2}\right)p(x_{1})$$

where the conditional probability

(12) $$p(x_{1}|x_{2})=N_{2|1}\exp\left(-\gamma_{2}/{\text{Q}}_{2}{\left(x_{2}-f(x_{1})\right)}^{2}\right),$$

and *p*(*x*_1_) is strongly peaked, as expressed e.g., in Equation (9) and Figure 3A. While Equation (11) is the standard formula of probability theory, Equation (12) is our specific result, which is derived from a Fokker-Planck equation (Haken, 2004, pp. 202–204). In the present context, Q_2_ represents the effect of random influences on 2, the patient. Large Q_2_ entails small Q^−1^_2_ ~ α in Equation (4), meaning high variance of self-states, i.e., volatility and little resilience.

An interesting consequence for the therapist is this: It makes sense in therapy to curb Q_2_, e.g., by strengthening the self-efficacy and the resilience of a patient. *f*(*x*_1_) in Equation (12) means shifting the patient's distinction-participation attractor of *x*_2_ (from a former *x*_2_ = β_2_) to a new value *x*_2_ = *f*(*x*_1_) fixed by the therapist. In the present context, the explicit form of *f*(*x*_1_) is not needed because the therapist just has to give it a special value (which, according to Equation (9) depends on his/her stable attractor *x*_1_ = β_1_).

While in physical systems (in thermal equilibrium) a fixed relation between γ_2_ and Q_2_ holds, this is not so in self-organizing systems. In psychological systems, we may assume that γ and Q can be chosen independently. Then person 1 (the therapist) is characterized by γ_1_ small, Q_1_ very small, so that $\alpha =\gamma_{1}/{\text{Q}}_{1}\to \infty$ (the therapist is immune to the impact of perturbations). Individual 2 (the patient) is characterized by γ_2_ large (willingness and/or capability of adaptation). γ_2_ is maintained or even strengthened by therapy, while Q_2_ is originally large. The task is to enhance $\gamma_{2}/{\text{Q}}_{2}$.

The outcome of therapy concerns the effect on person 2's potential, *V*_2_(*x*_2_). From Equations (1, 11, 12, 9) follows $\ln N-{\text{Q}}_{2}^{-1}V_{2}(x_{2})=\ln p(x_{2}|x_{1})=\ln N_{2|1}-\gamma_{2}/{\text{Q}}_{2}{\left(x_{2}-f(x_{1})\right)}^{2}$, so that

(13) $$V_{2}=const+\gamma_{2}{\left(x_{2}-f(x_{1})\right)}^{2}\text{ with }f(x_{1})=x_{2,new}\text{ fixed}$$

Clearly, the effect of therapy is a shift of the position of the minimum of *V*_2_, i.e., a new stable attractor in the patient's distinction/participation. This reflects new attitudes and/or behavior patterns. One may add that, in addition to the shifting of *x*_2_, a further goal of therapy is to lower Q_2_ toward the end of therapy and thus to increase the patient's resilience. Resilience is essential to preserve therapeutic gains post-therapy, and to prevent the patient's returning to old attractors in response to stressful live events.

## Discussion

We have initiated this paper with an introduction to the current focus of psychotherapy research on common factors. The most frequently discussed single factor is the therapeutic alliance. We explored this factor from three different perspectives: The first-person/experiential perspective provides subjective data, the third-person/behavioral perspective objective data, and, importantly, the structural/dynamical systems perspective models both kinds of data in an abstract way. We believe that the latter structural view is a significant amendment to the theory of psychotherapy because it promises to bridge the duality of subjective vs. objective approaches. Both subjective and objective data sources are valuable and indispensable, but they are much supported by an additional structural scaffold and generator of hypotheses, which we have shown can be provided by enactivist ideas that are then framed in the structural formalism of synergetics.

Before we discuss implications of the structural model presented above, we wish to address limitations of our treatment of the therapeutic alliance. An important aspect that we have not discussed in this article is the role of embodiment (*Leiblichkeit* in phenomenology, the material self of James, 1890). Embodiment is emphasized by the enactive approach to the self, which determines the status and role of the body more explicitly. The body is both the mediator and the matrix of social relationship and interaction. It grounds a sense of identity as separate (distinction), but also allows the person to be affected by the other (participation). The delevolped styles of engagement and qualities of interaction are inscribed in interactants' bodies, thus determining their current patterns of interaction. An important goal of future research will be to investigate how subjects co-negotiate their selves through bodily mediated engagements and how this is experienced in terms of a bodily sense of self. A further essential aspect is that a self is self-referential: Tschacher and Rössler (1996). We also did not explicitly cover the idea that the self is an emergent structure. Thus several further aspects of the self are discussed in the philosophical and psychological literature on consciousness and the self. The present considerations are not a full-fledged theory of the self, but they do provide a “minimal model” of therapeutic alliance that can be used as a heuristics and as a basis for further investigations.

We are aware that our use of enactivist, structural, and mathematical concepts may appear unusual to psychotherapy researchers and especially clinicians. Hence, it is all the more important to tie our approach to empirical evidence in the field of psychotherapy, and to show that new hypotheses and explanations may arise from it. We now sketch some of these empirical and practical implications in the following remarks.

### Complexity reduction in alliance formation

**Figure 5:**
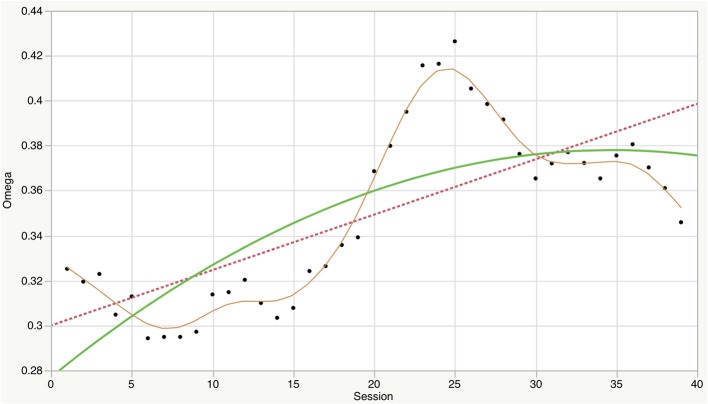
**Omega, a measure of system order (the inverse of complexity), was measured in a moving window throughout an exemplary therapy of 59 sessions**. Linear trend, quadratic trend, and a spline approximation (λ = 10) are shown as dashed, green, and brown lines. The data illustrate the overall order increase (reduction of complexity) in the course of this psychotherapy.

We have stressed the idea that alliance formation is crucial for change to occur in psychotherapy. This was illustrated by the geometrical phase space models (Figure 4) and expressed by the joint probability distribution of Equation (6) and its elaboration by the slaving principle of synergetics in Equation (11). Such alliance formation processes were observed in empirical studies, which showed that functional alliance is not generated by the “summation” of the participating individual systems, i.e., by the multiplication of individual probability distributions as in Equation (7). Instead, a novel system with interactive autonomy is formed during social interaction processes. This is what some enactive theorists would refer to as “participatory sense-making” (De Jaegher and Di Paolo, 2007). Without going into detail here, we may mention that numerous empirical findings speak for a reduction of complexity during the formation of therapeutic relationships (Tschacher et al., 2007). Reduced complexity means higher order and organization of the observed behavior due to the presence of attractors in alliance phase space. Figure 5 depicts how order changed in a single, exemplary therapy course. This psychotherapy of 59 sessions—which allowed computation of 39 consecutive omega values covering the complete course of therapy—was a Rogerian, client-centered therapy conducted at the outpatient psychotherapy clinic of the University of Bern. The patient was a woman aged 33 years. Her treatment entailed a larger than average symptom reduction (above the 75th percentile of the symptom checklist of the cohort at that time: Tschacher et al., 1998). The outcome in this therapy was especially favorable with respect to the social and phobic anxiety and depression symptoms of this patient.

Significant complexity reduction was also consistently found in empirical studies where the MEA methodology, mentioned in Section Introduction, was used (Tschacher et al., 2014a). Verbal, non-verbal, and other synchronies (e.g., based on judgments and meaning) indicate that the individuals are now parts of a coupled system. Coupled systems, especially non-linearly coupled systems, cannot be reduced to their components—a novel structure has emerged.

### Alliance is a special relationship

Therapeutic alliance is a special, asymmetrical relationship. In any social interaction, individuals have to negotiate their own self-maintenance in particular ways. There are some social interactions, however, that become more relevant than others to the individual's ability for preservation and organization of the self. The therapeutic alliance is one example for such a special relationship because it provides the patient with an opportunity for forming a relationship that is expected to not only perturb the patient's self-maintenance but also to foster and develop the patient's self-maintenance in the first place. That said, we see that the therapeutic alliance is an asymmetrical social relation because the focus of the interactions is on the patient, and the very goal of forming the relationship is to improve the patient's self-maintenance and not the therapist's (Equations 8, 9). In this, the patient-therapist alliance resembles an infant-caregiver relationship.

In the therapeutic alliance the therapist's phase space has become part of the patient's phase space offering calibration opportunities in regions that a “normal” joint phase space would potentially offer, but that the patient cannot reach by him-/herself because of developed attractors and meta-stable attractor ruins (Tsuda, 2013). In terms of the enactive approach this is to say that the therapist, instead of evaluating the social interaction with regards to his/her own self-maintenance (by the negotiation of distinction/participation), evaluates it with regards to the patient's self-maintenance. One might say that the two individuals effectively form a new autonomous identity whose self-organization relies on the contribution of two individuals but at the same time refers to only one of them (the patient). The therapist “offers” his/her striving for autonomy to the patient engaging in the interaction as an autonomous subject but at the same time evaluates the interactions heteronomously as contributions to the autonomy of the patient.

This also explains why authenticity is so important: the therapist must be both able to provide experiences of connection and of separation; if the therapist however acts “superior” or detached and not as a proper subject, the patient cannot experience the sense of open-ness required for developing a balanced distinction/participation. The therapist experiences the social interaction but reacts in it in a way as to accommodate the agreed goal of the alliance, that is to assist the patient's self-maintenance. The therapist lends him-/herself as a subject to build up a new identity for the patient; the tool for this is the interactive autonomy of the alliance.

### Temporality

The temporality of the therapeutic alliance is essential in several respects. Quite fundamentally, time scales play an important role as a key premise of self-organization processes. The two participants of the alliance have different relaxation times (Equation 10), which is the basis of the slaving principle. Therapists therefore have deeper and steeper attractors, see Equation (9), which they can employ to shift the location of patients' attractors and thus achieve a desired outcome.

Timing is also important because therapy courses pass through different stages. The therapy course shown in Figure 5 is illustrative in this respect because it shows that the order of the alliance often reaches a maximum at a time prior to the termination of therapy. Thus, later in the therapy course, the therapeutic bond has to dissolve, which can be measured by indicators of order and complexity.

### Corrective experience

In the formation of alliance, the patient's phase space is augmented through the therapist's phase space in that the therapist enables the patient to undergo “corrective emotional experiences,” one of the acknowledged common factors of psychotherapy (Tschacher et al., 2014b). This modifies the attractor landscape and allows the system to shift to formerly unattainable regions and to develop new trajectories and attractors (Equation 13). The therapist provides attractors to the alliance landscape that the patient cannot provide and does not (yet) have.

There are three points to be considered in the provision of corrective experiences. The first is the ability of the therapist to provide the patient with an augmented attractor landscape—can the therapist be a subject who offers both experiences of distinction and participation while not evaluating them with regards to him-/herself but to the patient? In other words, can therapist and patient form a new system by merging their individual phase spaces into one? It should be noted that it is only a partial fusion as the therapist is not to evaluate the outcome of the merging interaction with regards to him-/herself.

The second point is the potential of the interaction dynamics to gradually enable the patient to maneuver the augmented attractor landscape according to the goals of the patient's self-maintenance. This requires a flexibility and increased awareness on side of the therapist who has to provide the missing attractors/repellors when needed and/or when it is possible.

A third, and very crucial point is whether the alliance provides the patient with the capacity to dwell in his/her own new attractors in a way that does no longer involve the intervention and scaffolding provided by the therapist. The ultimate goal of a successful alliance is that the joint phase space becomes properly incorporated into the self-maintenance structure of the patient so that the therapist's contribution becomes obsolete. The therapist therefore has to enable the patient, by curbing Q_2_, to develop and rely on own abilities. In other words, the successful alliance allows the patient to interact with the therapist without staying emotionally dependent. Alliance dissolution is a major goal of psychotherapy when the change work is done.

### Outlook

We formulated a minimal model of therapeutic alliance starting from a one-dimensional concept of the psychological self with the poles distinction and participation. It will be a task of extended structural modeling to capture further aspects of the self (the agentic, embodied, self-referential, emergent self) in more complex models or through case-by-case analyses of the presented minimal model.

For empirical psychotherapy research, the present model generates a number of testable hypotheses, which to our knowledge are novel in psychology. The model claims, in Equation (10), that therapist and patient variables show markedly divergent relaxation times. The divergence of relaxation times leads to the slaving principle, a core proposition of “microscopic” synergetics (Haken, 2004), which provides a directed hypothesis for psychotherapy research: Successful therapists have much longer relaxation times than their patients. Ensuingly, some effort should be invested in exploring appropriate psychological indicators and measures of relaxation time, a psychometric task that appears to be resolvable. Enhancing $\gamma_{2}/{\text{Q}}_{2}$, as we stated above, is the goal during therapy: It can be achieved by patient's short relaxation time (which means high relaxation constant γ_2_) and high resilience against stressful perturbations (which means small Q_2_).

The testing of the core premise of our structural model—via the relaxation times and resilience of therapist and patient—could serve as an elegant cross-validation of previous “macroscopic” findings that psychotherapy systems as a whole self-organize, leading to the complexity reductions mentioned above in Section Complexity Reduction in Alliance Formation. Patient's resilience as a target of intervention is quite familiar to psychotherapy—one may think of stress inoculation training in cognitive behavioral approaches, which enhance Grawe's common factor “coping with the problem” (Meichenbaum, 1977). If, in addition, relaxation times prove to have favorable psychometric properties, a number of practical applications will come within sight. These applications may lead to feedback systems and innovations in therapist training, both major topics in current psychotherapy research (cf. Lutz et al., 2009; Taubner et al., 2013): A measure of relaxation time could be installed as a feedback device to predict positive outcome based on the dynamics of the therapeutic alliance, and therapists' training may in the future include interventions into own and patients' relaxation times.

### Conflict of interest statement

The authors declare that the research was conducted in the absence of any commercial or financial relationships that could be construed as a potential conflict of interest.
